# Assessing hippocampal functional reserve in temporal lobe epilepsy: A multi-voxel pattern analysis of fMRI data

**DOI:** 10.1016/j.eplepsyres.2013.01.004

**Published:** 2013-07

**Authors:** Heidi M. Bonnici, Meneka Sidhu, Martin J. Chadwick, John S. Duncan, Eleanor A. Maguire

**Affiliations:** a Wellcome Trust Centre for Neuroimaging, UCL Institute of Neurology, 12 Queen Square, London WC1N 3BG, United Kingdom; b Department of Clinical and Experimental Epilepsy, UCL Institute of Neurology, Queen Square, London WC1N 3BG, United Kingdom; c Epilepsy Society, Chalfont St Peter, Buckinghamshire, United Kingdom

**Keywords:** Epilepsy, Episodic memory, MVPA, Hippocampus, Hippocampal sclerosis, Functional MRI

## Abstract

Assessing the functional reserve of key memory structures in the medial temporal lobes (MTL) of pre-surgical patients with intractable temporal lobe epilepsy (TLE) remains a challenge. Conventional functional MRI (fMRI) memory paradigms have yet to fully convince of their ability to confidently assess the risk of a post-surgical amnesia. An alternative fMRI analysis method, multi-voxel pattern analysis (MVPA), focuses on the patterns of activity across voxels in specific brain regions that are associated with individual memory traces. This method makes it possible to investigate whether the hippocampus and related structures contralateral to any proposed surgery are capable of laying down and representing specific memories. Here we used MVPA-fMRI to assess the functional integrity of the hippocampi and MTL in patients with long-standing medically refractory TLE associated with unilateral hippocampal sclerosis (HS). Patients were exposed to movie clips of everyday events prior to scanning, which they subsequently recalled during high-resolution fMRI. MTL structures were delineated and pattern classifiers were trained to learn the patterns of brain activity across voxels associated with each memory. Predictable patterns of activity across voxels associated with specific memories could be detected in MTL structures, including the hippocampus, on the side contralateral to the HS, indicating their functional viability. By contrast, no discernible memory representations were apparent in the sclerotic hippocampus, but adjacent MTL regions contained detectable information about the memories. These findings suggest that MVPA in fMRI memory studies of TLE can indicate hippocampal functional reserve and may be useful to predict the effects of hippocampal resection in individual patients.

## Introduction

Hippocampal sclerosis (HS) is a common substrate for refractory focal epilepsy (Salmenpera and Duncan, 2005). Surgical resection carries a 60–70% chance of an individual being seizure free and having improved quality of life (Wiebe et al., 2001; deTisi et al., 2011) but brings cognitive risks, particularly to language and memory, if structures in the hemisphere contralateral to surgery are not fit for purpose (Scoville and Milner, 1957; Baxendale, 1998). For many years the Wada test (or intracarotid amobarbital procedure; Wada, 1949) was employed to help predict the risk of language impairment and amnesia following temporal lobe resection. However, the invasive nature of the Wada test and its methodological short-comings have prompted a significant decline in its use in recent years (Loring et al., 1992; Baxendale, 2000; Baxendale et al., 2005). In parallel, non-invasive functional MRI (fMRI) has been increasingly deployed to aid pre-surgical assessment of language dominance and memory (Baxendale, 2002; Rausch, 2002; Binder et al., 2008; Powell et al., 2008; Duncan, 2009).

fMRI is a reliable means of lateralising expressive language dominance in pre-surgical temporal lobe epilepsy patients (Baxendale, 2002; Salmenpera and Duncan, 2005; Duncan, 2009). However, assessing the functional integrity and reserve of critical memory structures such as the hippocampus is more difficult (Baxendale, 2002; Loring, 2005; Jones-Gotman, 2005; Jones-Gotman et al., 2008; Baxendale and Thompson, 2010; Bonnelli et al., 2010). This partly stems from the mass-univariate approach that is typically employed in fMRI studies. This involves creating a model of the experimental design that is fitted to the fMRI BOLD response at each voxel independently, the aim being to find activity in voxels that consistently shows a relationship with the experimental design, averaged over numerous trials. This method is highly effective for investigating many types of brain function, but is generally insensitive to fine-grained levels of representation such as individual memory traces. Thus, in the case of memory, one cannot know for certain that activation truly signals a brain region's capacity to represent individual memories.

In recent years an alternative analysis approach has emerged which exploits the intrinsically multivariate nature of fMRI data. This is motivated by the view that there may be information present in the distributed patterns of activation across voxels that is missed when looking at each voxel independently as in the mass-univariate method (Haynes and Rees, 2006; Norman et al., 2006). This multivariate approach is commonly known as multi-voxel pattern analysis (MVPA), or decoding. It involves training a support vector machine (SVM) classifier to recognise the patterns of activity across voxels associated with particular stimuli (e.g. specific memories). The classifier is then applied to a previously unseen portion of the data that was not used for training. If the classifier is successful at predicting (or decoding) which particular memory was being recalled in this test data set, this indicates that there is information about that memory represented in the brain region where the pattern of voxels was successfully identified. MVPA therefore affords the opportunity to examine the neural substrates of specific memory traces in individual participants, localised to brain regions of interest. Recent studies in healthy subjects have documented the power of this method for investigating neural information at the level of individual memory representations (reviewed in Rissman and Wagner, 2012). A number of these studies specifically focused on the hippocampus and surrounding medial temporal lobes (MTL) and successfully interrogated their capacity to represent various types of memory (Hassabis et al., 2009; Chadwick et al., 2010, 2011, 2012; Bonnici et al., 2012).

The ability to establish the functional capacity of the contralesional medial temporal lobe to sustain specific memories on an individual patient level has clear implications for surgical planning. Here we describe a novel approach of decoding specific neural signatures of memory representations within the medial temporal lobes using MVPA-fMRI in patients with TLE. Initially, we sought to ascertain whether this approach would provide results that were concordant with established clinical features in individuals with well-defined unilateral HS and memory profiles concordant with the laterality of their pathology. If MVPA has potential utility in the context of TLE, we would expect the classifier operating on voxels in the sclerotic hippocampus to perform at chance, as viable memory traces are unlikely to be laid down there. By contrast, the contralesional hippocampus should contain memory representations that a classifier can detect.

We employed a paradigm devised by Chadwick et al. (2010) in which participants viewed three short movies of everyday events prior to scanning, and then recalled them during high-resolution fMRI. This task had a number of advantages for our purpose; the memories involved were realistic and close to the experiences of day-to-day life, all participants were exposed to the same events permitting greater experimental control, and the task was simple and suitable for a range of abilities. Most importantly, this task revealed that decodable information about the memories was present bilaterally within the hippocampi, entorhinal/perirhinal and parahippocampal cortices in healthy subjects, making it suitable for use in individuals with both left and right HS. We therefore tested the hypothesis that the contralesional hippocampus and MTL would show the same pattern of findings as in the healthy controls (Chadwick et al., 2010) while results for the sclerotic hippocampus would be at chance.

## Methods

### Participants

This study was approved by the National Hospital for Neurology and Neurosurgery and the Institute of Neurology Joint Research Ethics Committee, and written informed consent was obtained from all participants, in line with the Declaration of Helsinki. Ten patients (8 female; 9 right handed) with a median age of 41 years, interquartile range 16.75, took part (Table 1). All had long-standing medically refractory TLE associated with unilateral HS, which had been identified on 3T structural MRI scans following qualitative assessment by expert neuroradiologists and quantification of hippocampal volumes and T2 relaxation times showing unilateral HS and apparently normal contralateral MTL structures. Nine individuals had left HS, and one had right HS, and all were taking anti-epileptic medication at the time of the study.

Neuropsychological assessment (Table 1) showed that their overall intellectual level was low average or average (WASI-III; Wechsler, 1997). Three patients with left HS had some mild word-finding difficulties (Graded Naming Test; McKenna and Warrington, 2007), but all could read proficiently and readily comprehended and complied with the task instructions. Executive function (The Weigl Colour-Form Sorting Test; Weigl, 1927) and visual perception (subtests from the Visual Object Space Perception Battery; Warrington and James, 1991) were within the average range. Memory (recall and recognition; visual and verbal) was assessed using several British-normed tests: the BMIPB (Coughlan et al., 2007), the Camden Memory Tests (Warrington, 1996), and the Recognition Memory Tests (Warrington, 1984). In general memory scores echoed the laterality of pathology, i.e. impaired or weaker verbal compared to visual memory in patients with left HS, and the opposite pattern in the patient with right HS (Table 1).

Our main interest was in comparing the affected MTL with the contralesional MTL within subjects, with patients effectively acting as their own controls. In this way it was possible to control completely for factors such as age, IQ, seizure history and medication regimen. However, in order to establish how the patients performed more generally, we also compared their fMRI data to a group of healthy control participants (also *n* = 10; six female; mean age 21.1 years, SD 1.8) who had performed the same task, in the same scanner using the same scanning parameters, and data analysis method. These control participants also gave informed written consent to participation in accordance with the local research ethics committee, and their data have been reported previously (see Chadwick et al., 2010).

### Pre-scan training

**Figure 1:**
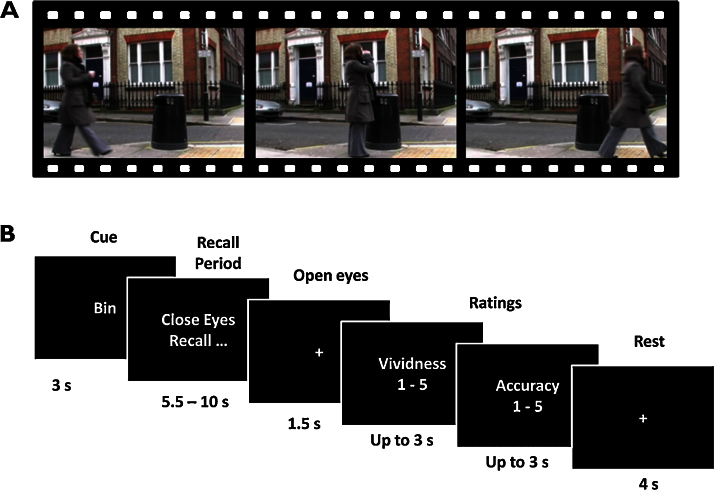
The experimental protocol. (A) Still photographs taken from one of the film clips viewed during pre-scan training. The clip depicted a woman taking a drink from a disposable coffee cup and then putting it in a bin (trashcan). (B) Timeline of an example trial during fMRI scanning.

The experimental protocol was the same as that employed by Chadwick et al. (2010), and the key features are reprised here for convenience. During a pre-scan training period, a patient viewed three film clips of everyday events. Each clip was 7 s long and featured a woman (a different one in each clip) carrying out a short series of actions. The films were shot outdoors in three different urban settings. These stimuli ensured that memories would be episodic-like in nature, and that all participants recalled the same set of memories. One clip featured a woman taking a letter out of her handbag, posting it in a post box (mailbox), and then walking off. Another clip featured a woman taking a drink from a disposable coffee cup, putting the cup in a rubbish bin (trashcan), and then walking off (Fig. 1A). The final clip featured a woman picking up a bicycle that was leaning against some railings, adjusting her helmet and walking off with the bicycle. Patients viewed each clip 15 times and practiced recalling them as vividly as possible and within the 7 s timeframe. Patients were also trained on a rating procedure where they evaluated each recall period for the level of vividness as well as how consistent the recall had been relative to previous recollections of the same memory. This practice period therefore ensured that the memory traces for the three movies were stable before going into the scanner, a prerequisite for the subsequent MVPA analysis.

### Scanning task

During scanning patients recalled each movie twenty times in a pseudo-random order, ensuring that the same memory was not repeated two or more times in a row. On each trial (see Fig. 1B) the patient viewed a cue on screen indicating which of the three film events they should recall. Patients were instructed not to begin the recall process until the cue to close their eyes and recall appeared. To ensure the patients were concentrating, and that the recall approximated the original 7 s length of a clip, the patient had to press a button (using a scanner-compatible button-box) when they had finished recalling the clip. If the button was pushed too soon (<5.5 s) or they failed to push it within 10 s then a tone would sound, and a message would appear for 1.5 s indicating that their recall had been too fast or too slow. Any such trials were excluded from the subsequent analysis. If the patient pressed the button between 5.5 and 10 s, a fixation cross appeared onscreen for 1.5 s. Patients were trained to open their eyes as soon as they had pressed the button or if they heard the tone. Following this, the patient was required to provide ratings about the preceding recall trial using the button-box, just as they had been trained to do during the pre-scan training session. First, they rated how vivid the preceding recall trial was (scale: 1–5, where 1 was not vivid at all, and 5 was very vivid). Second, they rated how accurately the recalled memory reflected the actual film clip (scale: 1–5, where 1 was not accurate at all, and 5 was very accurate). Any trials where a participant recorded a rating of less than 3 were excluded from the subsequent analysis. Following the ratings, participants rested for 4 s before starting the next trial. Once excluded trials were discounted (and similar to the control data in Chadwick et al., 2010), this resulted in an average of 12.3 (SD 1.34) trials for memory 1, 12.5 (SD 2.42) trials for memory 2 and 12.9 (SD 1.97) trials for memory 3 making an average of 36 trials in total per patient being entered into the MVPA analysis.

### Data acquisition

Using high-resolution fMRI (Carr et al., 2010), we acquired data in a limited volume focussing on the medial temporal lobe, particularly the hippocampus entorhinal/perirhinal and posterior parahippocampal cortices. A 3T Magnetom Allegra head only MRI scanner (Siemens Healthcare, Erlangen, Germany) operated with the standard transmit-receive head coil was used to acquire the functional data with a T2*-weighted single-shot echo-planar imaging (EPI) sequence in a single session (in-plane resolution = 1.5 mm × 1.5 mm; matrix = 128 × 128; field of view = 192 mm × 192 mm; 35 slices acquired in interleaved order; slice thickness = 1.5 mm with no gap between slices; echo time TE = 30 ms; asymmetric echo shifted forward by 26 phase-encoding (PE) lines; echo spacing = 560 μs; repetition time TR = 3.5 s; flip angle *α* = 90°). All data were acquired at 0° angle in the anterior-posterior axis. An isotropic voxel size of 1.5 mm × 1.5 mm × 1.5 mm was chosen for an optimal trade-off between BOLD sensitivity and spatial resolution. Further, the isotropic voxel dimension reduced re-sampling artefacts when applying motion correction. To ensure optimal data quality, images were reconstructed online and underwent online quality assurance (Weiskopf et al., 2007). For distortion correction (Hutton et al., 2002), field maps were acquired with the standard manufacturer's double echo gradient echo field map sequence (TE = 10.0 and 12.46 ms, TR 1020 ms; matrix size, 64 × 64), using 64 slices covering the whole head (voxel size 3 mm × 3 mm × 3 mm). In addition to the functional scans, a whole brain T1-weighted 3D FLASH sequence was acquired with a resolution of 1 mm × 1 mm × 1 mm. A T1-weighted high-resolution 3D modified driven equilibrium Fourier transform whole-brain structural MRI scan was acquired for each patient after the main scanning session with 1 mm isotropic resolution (Deichmann et al., 2004).

### Region of interest (ROI) segmentation

**Figure 2:**
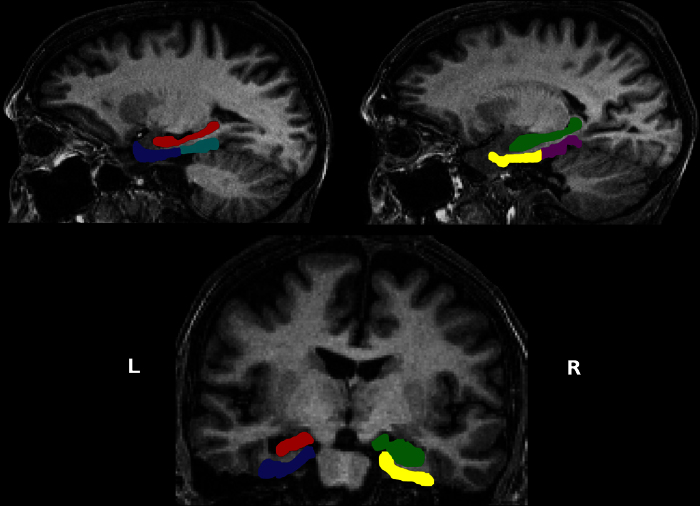
Brain regions of interest. The top panel shows sagittal views through the left and right hemispheres, and the lower panel a coronal section, from the structural MRI scan of one of the patients with left HS; L = left side of the brain, R = right side of the brain. The left hippocampus (sclerosed) is coloured in red, the right hippocampus in green, the left EPC in blue, the right EPC in yellow, the left PHC in turquoise, and the right PHC in magenta. (For interpretation of the references to colour in this figure legend, the reader is referred to the web version of the article.)

Given our specific interest in the hippocampus and medial temporal lobes, manual segmentation of the hippocampus (HC), entorhinal/perirhinal cortex (EPC, combined) and parahippocampal cortex (PHC) was performed using ITK-SNAP (Yushkevich et al., 2006 – www.itksnap.org) using the 1 mm × 1 mm × 1 mm T1 structural images (see example in Fig. 2). Hippocampal anatomy was identified using the Duvernoy hippocampus atlas (Duvernoy, 2005). The entorhinal/perirhinal cortex and parahippocampal cortex were segmented according to the protocol described by Insausti et al. (1998). Author HMB performed the segmentations, and inter-rater reliability was calculated on a random selection of four patient scans which had also been segmented by author MS. The Dice overlap metrics (Dice, 1945) were as follows: HC 0.81, EPC 0.63, PHC 0.60. Mean volumes (in cubic mm) and standard deviations (SD) for the ROIs in the left (L) and right (R) MTL in the group of nine LHS patients were as follows: HCL 2435.11 (SD 467.94), HCR 3415.89 (453.01), EPCL 3085.33 (453.01), EPCR 3159.33 (326.71), PHCL 1591.22 (386), and PHCR 1543.56 (272.99). A repeated measures ANOVA revealed a significant hemisphere by region interaction (*F* = 24.37, *p* < 0.0001). Further investigation of this effect using paired *t*-tests confirmed that a volume difference between the left and right hippocampus drove this result (*t* = −6.06, *p* < 0.0001) with the right hippocampal volume greater than the left. There were no differences in volume between the left and right sides for the other MTL structures (EPC: *t* = −1.07, *p* = 0.32; PHC: *t* = 0.42; *p* = 0.69). For the patient with right HS, the hippocampal volume difference was also apparent, but with the left greater than the right: HCL 3503, HCR 2059, EPCL 2569, EPCR 2956, PHCL 1297, and PHCR 1433.

### Image pre-processing

Pre-processing of the fMRI data was performed using SPM8 (www.fil.ion.ucl.ac.uk/SPM). The first six EPI volumes were discarded to allow for T1 equilibration effects (Frackowiak et al., 2004). The remaining EPI images were then realigned to correct for motion effects, and minimally smoothed with a 3 mm FWHM Gaussian kernel. A linear detrend was run on the images to remove any noise due to scanner drift (LaConte et al., 2005). Next the data were convolved with the canonical haemodynamic response function (HRF) to increase the signal-to-noise ratio (Frackowiak et al., 2004). This HRF convolution effectively doubled the natural BOLD signal delay, giving a total delay of approximately 12 s. To compensate for this delay, all onset times were shifted forward in time by three volumes, yielding the best approximation to the 12 s delay given a TR of 3.5 s and rounding to the nearest volume (Duda et al., 2001). Functional volumes were extracted from the vivid recall period of each trial, leading to a total of four functional volumes per trial.

### Multi-voxel pattern analysis

A support vector machine classifier was created for each region of interest. Each classifier was trained on a portion of the fMRI data relating to the three episodic memories and then tested on an independent set of instances of these memories. We used a standard MVPA procedure that has been described elsewhere (Chadwick et al., 2010; Bonnici et al., 2012; for an in-depth review of MVPA in the hippocampus see Chadwick et al., 2012). Briefly, the overall classification procedure involved splitting the fMRI data into two segments: a “training” set used to train a classifier with fixed regularisation hyperparameter *C* = 1, in order to identify response patterns related to the memories being discriminated, and a “test” set used to independently test the classification performance (Duda et al., 2001), using a cross-validation procedure. We used a standard *k*-fold cross-validation testing regime wherein *k* equalled the number of experimental trials, with the data from each trial set aside in turn as the test data, and the remaining data used as the training set. This therefore generated *k* sets of SVM training and test sets which produced an overall classification accuracy from the proportion of correct classification “guesses” across all *k* folds of the cross-validation. The classification was performed using the LIBSVM implementation (Chang and Lin, 2011).

Prior to multivariate classification, feature selection (Guyon and Elisseeff, 2003) was performed on the data from the training set (thereby ensuring that this step was fully independent from final classification, which is critical for avoiding “double-dipping” – Kriegeskorte et al., 2009). The purpose of feature selection is to reduce the set of features (in this case, voxels) in a dataset to those most likely to carry relevant information. This is effectively the same as removing voxels most likely to carry noise, and is a way of increasing the signal-to-noise ratio. This was conducted using a standard multivariate searchlight strategy within the given ROI. For a given voxel, we first defined a small sphere with a radius of three voxels centred on the given voxel (Kriegeskorte et al., 2006; Hassabis et al., 2009; Chadwick et al., 2010, 2012; Bonnici et al., 2012). Note that the spheres were restricted so that only voxels falling within the given region of interest were included. Therefore, the shape of the sphere and the number of voxels within it varied depending on the proximity to the region of interest's borders. This procedure then allowed the selection of the searchlight voxel set that contained the greatest degree of decoding information within the training dataset. Using this voxel subset, the SVM classifier was trained to discriminate between the three episodic memories using the “training” dataset, and tested on the independent “test” dataset.

Standard SVMs are binary classifiers that operate on two-class discrimination problems, whereas our data involved a three-class problem (i.e. three memories). The SVM can, however, be arbitrarily extended to work in cases in which there are more than two classes. Typically this is done by reducing the single multiclass problem into multiple binary classification problems that can be solved separately and then recombined to provide the final class prediction (Allwein et al., 2000). We used the well-established Error Correcting Output Codes approach (Dietterich and Bakiri, 1994) and computing of the Hamming distance (Hamming, 1950; Hassabis et al., 2009; Chadwick et al., 2010, 2011).

### Data analysis

The classifier accuracy values for each brain region were compared to chance using one-tailed *t*-tests. Because there were three memories, chance was 33% in this study. Other within-subjects comparisons were made using repeated measures ANOVA and paired *t*-tests. We report formal statistical analyses for the group (*n* = 9) of left HS patients, and present the individual data of the right HS patient. We also compared the performance of the patients (*n* = 9 with left hippocampal sclerosis) with the 10 healthy participants reported by Chadwick et al. (2010) using ANOVA. A threshold of *p* < 0.05 was employed throughout.

## Results

For each brain region of interest (see “Methods” section) a classifier was first trained on a portion of the fMRI data relating to the three episodic-like memories and then tested on an independent set of trials of these memories. A classification result significantly above chance (33%) would be expected if information was present in the patterns of fMRI activity that enabled discrimination between the three memories. We performed this analysis for each ROI in both hemispheres.

**Figure 3:**
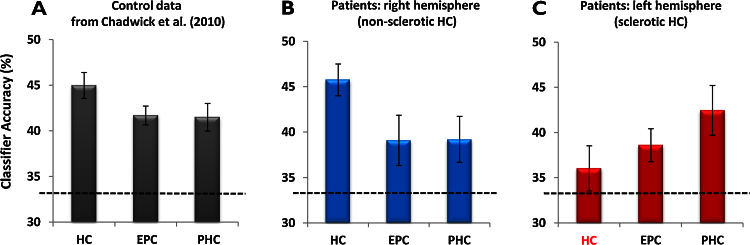
Results of the MVPA analyses. (A) Control data (*n* = 10, collapsed across hemispheres) from Chadwick et al. (2010) using the same task as that employed in the current study. Mean classifier accuracy performances (± one standard error of the mean) are shown; chance was 33% (represented by the black dashed line). HC = hippocampus; EPC = entorhinal/periphinal cortex; PHC = parahippocampal cortex. (B) Data for the contralesional right MTL (from the 9 patients with left HS). (C) Results for the affected left MTL, where classifier performance for the sclerosed hippocampus (highlighted in red on the *x*-axis) was not significantly different to chance. Classifier performances in the other left MTL regions and all areas in the contralesional MTL were significantly above chance. (For interpretation of the references to colour in this figure legend, the reader is referred to the web version of the article.)

In order to contextualise the current findings, it is worth noting that when using this paradigm in healthy subjects, Chadwick et al. (2010) found that all three MTL regions produced classification results that were significantly above chance, showing that it was possible to detect information about individual episodic-like memories from activity patterns within the hippocampus and surrounding MTL (see Fig. 3A). The results were highly similar for left and right MTL. They also found that classification accuracy for the hippocampus was significantly better than for the other MTL regions, suggesting that episodic-like memories may be more distinct within the hippocampus than the surrounding cortex.

In a similar vein, we found that in the patients, classifiers operating on the patterns of activity across voxels in each region of the contralesional MTL predicted which memory was being recalled significantly above chance (HCR: *t* = 7.299, *p* = 0.0001; EPCR: *t* = 2.206, *p* = 0.029; PHCR: *t* = 2.450; *p* = 0.020) (Fig. 3B). Classification accuracy was significantly higher in the hippocampus compared to the EPC (*t* = 2.551, *p* = 0.034; not significant compared to the PHC *t* = 1.788, *p* = 0.112). This shows that the episodic-like memories of the movies were represented in the hippocampus in particular, and also in adjacent MTL structures, suggesting operational memory capacity of these contralateral MTL regions.

By contrast, the classifier accuracy within the sclerotic left hippocampus for the three memories was not significantly greater than chance (HCL: *t* = 1.225, *p* = 0.128; Fig. 3C), while classifier performances in the surrounding MTL regions were significantly above chance (EPCL: *t* = 3.067, *p* = 0.008; PHCL: *t* = 3.444, *p* = 0.004). Classification accuracy was significantly higher in the parahippocampal cortex than the sclerotic hippocampus (*t* = −3.853, *p* = 0.005). This suggests that discernable memory representations were absent in the sclerotic hippocampus, but present in adjacent MTL structures.

Having examined MTL structures in each hemisphere separately, we then made direct comparisons using a repeated-measures ANOVA. There was a significant interaction between the two factors of hemisphere and region (*F* = 4.561; *p* = 0.026) that was driven by a significant difference in classifier performance between the sclerotic and contralesional hippocampi (*t* = −3.989, *p* = 0.004), with the classifier operating on voxels in the sclerotic left hippocampus unable to distinguish between the three episodic memories.

Eight of the nine left HS patients showed a difference in performance between the hippocampi in the predicted direction; the performance of the classifier operating on voxels in the contralesional right hippocampus was greater than that of the sclerotic left hippocampus (mean classifier performance HCL = 34.94%, SD = 7.16; HCR = 46.10%, SD = 5.51). Data for the one patient with right HS was also in the expected direction, with deceased classifier accuracy within the sclerosed right hippocampus (HCL 44.44%, HCR 36.67%). We also examined whether the at-chance result in the sclerotic hippocampi might have been associated with its smaller volume. No correlation between classification accuracy and hippocampal volume was found (Pearson *r* = 0.511, *p* = 0.16).

The ninth left HS patient's results were equivocal (patient 9: HCL = 44.95%, HCR = 43.12%; volumes for this patient: HCL 2970, HCR 3478). To investigate if this was related to clinical factors, correlations of classifier accuracy with variables such as patients’ current age, age of epilepsy onset, epilepsy duration and seizure frequency were performed. No significant correlations were observed, although this may be due to the relatively small sample size.

Finally, we directly compared the fMRI data from the nine left hippocampal sclerosis patients with that of the ten healthy control participants reported by Chadwick et al. (2010). Despite the controls being younger than the patients, there were no significant differences in classifier accuracies between the patient and controls for any MTL region except the left hippocampus, where classifier accuracy was significantly less in the patients (HCL *F* = 6.52, *p* = 0.02; HCR *F* = 0.001, *p* = 0.97; EPCL *F* = 1.57, *p* = 0.23; EPCR *F* = 0.94, *p* = 0.35; PHCL *F* = 0.7, *p* = 0.41; PHCR *F* = 1.3, *p* = 0.27). Of particular note, there was no difference between the groups in classifier accuracy for the right hippocampus. These findings therefore affirm the view that the sclerotic left hippocampi in the patients were dysfunctional, while the right hippocampi retained a normal level of mnestic functioning.

## Discussion

We found that predictable patterns of activity across voxels associated with specific memories could be detected in MTL structures, including the hippocampus, on the side contralateral to focal unilateral hippocampal sclerosis in a group of patients with TLE. By contrast, no discernible memory representations were apparent in the sclerotic hippocampus, although the adjacent MTL regions on that side contained detectable information about the memories. To our knowledge, this is the first MVPA-fMRI study to be reported in TLE, and therefore shows that the MVPA technique, designed to detect specific memory representations in patterns of fMRI data, permits interrogation of MTL functionality and in particular hippocampal functional reserve, complementing existing investigative protocols in TLE.

The paradigm we employed involved exposing all patients to the same set of movies that depicted everyday events. Memories of these naturalistic stimuli have been found to be encoded and represented in the left and right hippocampus of healthy controls, such that recall of these memories could be predicted from patterns of fMRI brain activity across hippocampal voxels (Chadwick et al., 2010). By capitalising on the bilateral nature of the memory traces associated with these episodic-like memories, we could examine and compare the memory capacity of the hippocampi in patients in whom one hippocampus was sclerotic. In this study we therefore deliberately focussed on patients where the structural imaging and neuropsychological tests pointed to selective involvement of one sclerotic hippocampus. That this damage compromised the mnestic capacity of that hippocampus was clear in the at-chance performance of the pattern classifier, which could not discern any reliable patterns of activity associated with the memories. This was in direct contrast to the contralateral hippocampus in which the memory traces were readily detected. Moreover, the contralateral hippocampus contained similar amounts of information about the memories when the patients were compared to healthy control participants, while the sclerotic hippocampus was severely compromised. Overall, therefore, the within- and between-subjects effects we observed suggest the MVPA approach is reliable, and offers new insights into hippocampal functional reserve in TLE.

This method was not only able to interrogate the functional status of the hippocampus, but also the surrounding medial temporal lobe tissue. The entorhinal/perirhinal and parahippocampal cortices in the contralesional hemisphere of the patients contained decodable information about the memories, and less so than the hippocampus, confirming the functional integrity, and functional hierarchy, of the wider MTL memory system on that side. Of particular note was the status of the EPC and PHC on the affected side, as information about the memories was detectable in these structures. This shows that in the presence of HS, adjacent MTL regions can retain a capacity to contribute to memory recall. The classifier operating on voxels in the PHC on the affected side seemed to perform better than its counterpart on the unaffected side (Fig. 3B and C), perhaps indicating a compensatory mechanism. However, when directly compared, there was no significant difference between the two (*t* = 0.97; *p* = 0.36), and no difference when compared to the control participants.

How do our MVPA findings relate to results from conventional fMRI memory studies in TLE? After all, asymmetric MTL activations have also been observed using standard fMRI paradigms, and much information can be gleaned from such studies. For instance, in tasks that were associated with bilateral activity within the MTL in healthy control participants, patients showed significantly reduced MTL activation ipsilesionally (e.g. Detre et al., 1998; Jokeit et al., 2001; Mechanic-Hamilton et al., 2009). In a complex scene encoding task seven patients with left TLE and three with right TLE were studied using a simple block design fMRI protocol. Patients showed asymmetry of activation with greater activation in the non-lesional MTL. However, fMRI activations were not correlated with neuropsychological performance, therefore it could not be definitively ascertained if contralateral activations were specifically related to successful memory encoding (Detre et al., 1998). Jokeit et al. (2001) employed a spatial navigation task in a group of thirty patients with refractory TLE. Asymmetry of activation was seen in 90% of patients. Rank correlation was performed between the number of activated voxels within the MTL, out of scanner neuropsychological variables, and Wada test hemispheric memory performance. Correlations between visuospatial memory performance and right MTL activation but not left MTL activation were found. Left MTL activation was correlated with Wada hemispheric memory performance for visually presented objects. These correlations suggested that MTL activations during the task were related to the memory encoding process.

These examples illustrate that conventional fMRI BOLD activations typically index the involvement of brain regions in an experimental task. However, to more directly link the engagement of a brain area to successful memory function, further correlation with neuropsychological variables (as in Jokeit et al., 2001) or subsequent memory paradigms using, for example, an event-related analysis is necessary (Powell et al., 2007). MVPA, however, offers the important added value of being able to assess the function of MTL structures at the level of fine-grained individual memory representations, which is a more direct reflection of the capacity of the MTL structures. This method provides the additional benefit of being able to distinguish specific neural representations within relatively small sub-regions of the medial temporal lobe in individual patients, thereby offering another dimension to delineating the functional topography of memory representations in the context of TLE.

Conventional fMRI memory encoding studies have also examined material-specific effects and some have observed memory reorganisation involving the contralesional MTL (Golby et al., 2002; Powell et al., 2007). In left TLE patients subsequent memory studies using an event-related analysis showed reorganisation of verbal memory encoding to the contralateral hippocampus (Richardson et al., 2003) and in right TLE patients reorganisation of visual encoding to involve the left hippocampus (Powell et al., 2007). It will be interesting for future studies to employ MVPA in this context to investigate the capacity for effective reorganisation in unilateral TLE.

In a similar vein, pre-operative conventional fMRI activations have been shown to be important predictors of post-surgical memory outcome in both material-specific memory paradigms (Bonnelli et al., 2010; Powell et al., 2008; Richardson et al., 2004) and tasks that are more bilaterally represented within the MTL (Janszky et al., 2005; Mechanic-Hamilton et al., 2009) in group studies. Because MVPA indexes the presence of predictable and useful information pertaining to specific memories, and given that we have shown evidence for functional capacity within the MTL even on a single subject basis, this leads to the exciting possibility that pre-operative MVPA could also predict post-operative memory change in patient groups but also, crucially, at the level of individual patients.

Overall, our findings indicate that MVPA-fMRI could prove a useful non-invasive method of assessing pre-surgical memory capacity within the MTL. Despite our small sample size, our results illustrate that the MVPA approach to fMRI memory studies in the context of TLE can give an insight into hippocampal functional reserve. While this MVPA-fMRI method for assessing memory reserve shows promise, our study reveals clear targets for future inquiries. Here we examined memory recall in individuals with clear-cut unilateral HS (mostly left-sided) and memory profiles that echoed the laterality of this pathology. This was the logical first step in evaluating MVPA-fMRI because if the results did not concur with known pathology, this would discourage its future use. As it is, MVPA may be useful not only in addition to existing structural and functional MRI and neuropsychological data in straightforward cases, but could play a particularly useful role in patients in whom existing information is ambiguous (Baxendale et al., 2005). In those situations, MVPA-fMRI could give insights into the memory reserve of the hippocampi and surrounding MTL structures that may have a direct bearing on surgical decisions. Further work is now required to assess the validity of the MVPA-fMRI approach in larger cohorts, and patients with unilateral left and right HS as well as more complex cases. It will also be important to explore the efficacy of MVPA-fMRI with other memory paradigms, to investigate the concordance of MVPA with clinical data, and in particular the link between the absence of pattern recognition at the level of the sclerotic hippocampus and post-surgical memory status.

Finally, MVPA as it is commonly implemented including its use here, involves training support vector machine classifiers. This requires multiple presentations of the same stimuli in order to accrue enough examples of the brain activity patterns associated with each stimulus for training to be viable. As such, the technique depends on brain activity being stable from one instance of stimulus presentation to the next. Certain types of design, therefore, become more challenging, including learning paradigms in which the neural signatures of stimuli may be more dynamic. However, decoding methods are constantly evolving (Chadwick et al., 2012) and it is likely that opportunities for new applications in the context of pre-surgical evaluation of TLE patients will arise.

## Conflict of interest

None of the authors has any conflict of interest to disclose.

## Figures and Tables

**Table 1:** Summary of patient details.

ID	Gender	Age (yrs)	H’ess	Cognitive summary^a^	Age at onset (yrs)	Duration (yrs)	MRI	Seizure type and frequency
1	F	41	R	IQ low average; verbal mem imp; visual mem average	32	9	LHS	CPS: 2–3/month; SGS: 2/month
2	F	51	R	IQ average; verbal mem average; visual mem above average	20	31	LHS	CPS: 1/year; SGS: 2 in the past
3	M	20	L	IQ average; verbal mem average; visual mem average	13	7	LHS	CPS: 4–8/month; SGS: 2/year
4	F	55	R	IQ average; verbal mem borderline imp; visual mem low average	14	41	LHS	CPS: 2/week; SGS: 2/year
5	F	42	R	IQ average; verbal mem imp; visual mem low average	11	31	LHS	CPS: 3–4/month
6	M	29	R	IQ average; verbal mem imp; visual mem average	6	23	LHS	CPS: 1/month; SPS: 1/month; SGS: 1/year
7	F	44	R	IQ low average; verbal mem imp; visual mem above average	18	26	LHS	Previously CPS: 2–3/month; currently controlled on AED
8	F	52	R	Chose not to attend for cognitive testing	12	40	LHS	CPS: 1/month; SGS: 1/year
9	F	50	R	IQ average; verbal mem imp; visual mem above average	35	15	LHS	CPS: 16/month; SGS: 2/year
10	F	37	R	IQ average; verbal mem low average; visual mem imp	2	35	RHS	CPS: 4–6/month; SGS S: 2/year

yrs = years; H’ess = handedness; R = right; L = left; imp = impaired; mem = memory; LHS = left hippocampal sclerosis; RHS = right hippocampal sclerosis; CPS = complex partial seizure; SGS = secondarily generalised tonic–clonic seizure; SPS = simple partial seizure; AED = anti-epileptic drugs.

a Impaired = <2nd percentile; borderline impaired = 2nd–9th percentile; low average = 10th–24th percentile; average = 25th–75th percentile; above average = 76th–90th percentile; superior = 91st + percentile. See “Methods” section for details of specific neuropsychological tests.
